# Comparison of new hyperspectral index and machine learning models for prediction of winter wheat leaf water content

**DOI:** 10.1186/s13007-021-00737-2

**Published:** 2021-03-31

**Authors:** Juanjuan Zhang, Wen Zhang, Shuping Xiong, Zhaoxiang Song, Wenzhong Tian, Lei Shi, Xinming Ma

**Affiliations:** 1 Collaborative Innovation Center of Henan Grain Crops, Henan Agricultural University, #63 Nongye Road, Zhengzhou, 450002 Henan People’s Republic of China; 2 College of Agronomy, Henan Agricultural University, #63 Nongye Road, Zhengzhou, Henan 450002 People’s Republic of China; 3 Science College of Information and Management, Henan Agricultural University, #63 Nongye Road, Zhengzhou, Henan 450002 People’s Republic of China; 4 Adelphi University, # One South Avenue, Garden City, NY 11530-0701 USA; 5 Luoyang of Agriculture and Forestry, #1 Nongke Road, Luoyang, 471000 Henan People’s Republic of China

**Keywords:** Winter wheat, Leaf water content, Spectral index, Characteristic band, Modeling method, Inversion model

## Abstract

**Background:**

The leaf water content estimation model is established by hyperspectral technology, which is crucial and provides technical reference for precision irrigation.

**Methods:**

In this study, two consecutive years of field experiments (different irrigation times and seven wheat varieties) in 2018–2020 were performed to obtain the canopy spectra reflectance and leaf water content (LWC) data. The characteristic bands related to LWC were extracted from correlation coefficient method (CA) and x-Loading weight method (x-Lw). Five modeling methods, spectral index and four other methods (Partial Least-Squares Regression (PLSR), Random Forest Regression (RFR), Extreme Random Trees (ERT), and K-Nearest Neighbor (KNN)) based characteristic bands, were employed to construct LWC estimation models.

**Results:**

The results showed that the canopy spectral reflectance increased with the increase of irrigation times, especially in the near-infrared band (750–1350 nm). The prediction accuracy of the newly developed differential spectral index DVI (R1185, R1307) was higher than that of the existing spectral index, with R^2^ of 0.85 and R^2^ of 0.78 for the calibration and validation, respectively. Due to a large amount of hyperspectral data, the correlation coefficient method (CA) and x-Loading weight (x-Lw) were used to select the water characteristic bands (100 and 28 characteristic bands, respectively) from the full spectrum. We found that the accuracy of the model based on the characteristic bands was not significantly lower than that of the full spectrum-based models. Among these models, the ERT- x-Lw model performed the best (R^2^ and RMSE of 0.88 and 1.46; 0.84 and 1.62 for the calibration and validation, respectively). In addition, the accuracy of the LWC estimation model constructed by ERT-x-Lw was higher than that of DVI (R1185, R1307).

**Conclusion:**

The two models based on ERT-x-Lw and DVI (R1185, R1307) can effectively predict wheat leaf water content. The results provide a technical reference and a basis for crop water monitoring and diagnosis under similar production conditions.

## Background

Wheat is the main food crop in North China. Due to the imbalance between precipitation and water demand during the growing period, reasonable irrigation has become a necessary condition for a high yield of wheat [1]. The leaf is an essential component of plant canopy structure and the site where a substantial number of important biochemical processes occur; thus, leaf water content is an important indicator that reflects the overall crop water status and indirectly indicates the input and output of soil water [2, 3]. Therefore, the leaf water content can be used as a reliable reference index for making feasible irrigation decisions [4]. Hyperspectral remote sensing technology has the advantages of being fast, economic, and nondestructive. It can be used to monitor the growth of crops by obtaining reflectance information. Hence, the development of a diagnostic model of water status by hyperspectral remote sensing technology is of substantial significance for precision irrigation and water-saving irrigation.

Currently, hyperspectral remote sensing technology is widely used in crop water monitoring scenarios. In previous studies, the relationship between wheat leaf water content and hyperspectral data has been analyzed, and the spectral index has been used to estimate leaf water content. For example, the leaf water content of rice, peanut, soybean, and wheat can be well predicted by the ratio of the water index to the normalized vegetation index (WI(R_900_/R_970_)/NDVI(R_900_ − R_680_)/(R_900_ + R_680_), where R is the wavelength of the original spectral reflectance) [5]. Zhao et al. [6] established the presence of a significant correlation between leaf water content and the normalized difference water index (NDWI), the simple ratio (SR), and the shortwave infrared perpendicular water stress index (SPSI). Moreover, Rapaport et al. [7] developed a water balance index (WABI = (R_1500_ – R_531_)/ (R_1500_ + R_531_)) for monitoring the plant water status in grapevine under field conditions. In recent years, machine learning methods have been increasingly utilized for modeling and analysis of wheat growth information and water index. In this respect, Zhang et al. [8] used reflectance data in the range of 859–1640 nm, partial least squares (PLSR), artificial neural network (ANN), and support vector machine (SVN) algorithms to construct models for estimating wheat leaf water content and the equivalent water thickness content, and compared the prediction accuracy with the vegetation index model, employed as a reference model. Additionally, Das et al. [9] established a prediction model of wheat relative water content using a PLSR algorithm, multiple linear regression, SVN and random forest regression (RFR), based on hyperspectral and relative water content data of 10 wheat varieties in different periods. When full-band data are used for modeling, some issues arise, such as interference of redundant band information and long operation time, caused by a large amount of data, which exerts a certain negative impact on the model accuracy [10]. Therefore, it is highly important to select sensitive bands related to agronomic parameters. Sun et al. [11] utilized continuous wavelet transform (CWT) to decompose and transform the canopy spectra under different irrigation treatments, and established that the PLSR model constructed using 2400, 1596, and 2397 nm bands effectively estimated wheat equivalent water thickness (EWT). In another investigation, Krishna et al. [12] found that the whole spectral reflectance band of rice was reduced to 32 by the loading weight method, which did not decrease the accuracy of the model. In addition to the aforementioned modeling methods, random forest, extreme random tree and k-nearest neighbor algorithms have also been used to estimate biomass, nitrogen content, leaf area, and SPAD [13–15]. However, these methods have been rarely applied for the assessment of wheat leaf water content.

In this study, five different models were established separately based on the canopy reflectance of seven wheat varieties under different irrigation treatments from 2018 to 2020. The main purpose of this investigation was to provide a future reference for hyperspectral monitoring of winter wheat leaf moisture under similar production conditions. To achieve this purpose, three major experimental sub-goals were defined: (i) to analyze the effect of different irrigation times on wheat leaf water content and spectral reflectance; (ii) to evaluate the performance of newly developed spectral indices and existing spectral indices for leaf water content; and (iii) to compare the ability of four the regression models established by the four algorithms to monitor leaf water content and to identify the optimal model among them.

## Experimental materials and methods

### Design of field experiments

#### Experiment 1

The experiment was conducted in the field of Luoyang Academy of Agricultural and Forestry Sciences, Luoyang City, Henan Province, China (112° 49′ 18″ E, 36° 64′ 14″ N from 2018 to 2019). The soil type is loamy cinnamon soil (Organic Matter: 14.4 g kg^−1^, Total N:1.83 g kg^−1^, P_2_O_5_:24.6 mg kg^−1^, K:126.9 mg kg^−1^). The experimental variables included different irrigation period treatments (w0: irrigation at the bottom stage w1: irrigation at the bottom and jointing stage, w2: irrigation at bottom, jointing and grain-filling stage) and seven different winter wheat cultivars (Luomai 27, Zhengmai 136, Zhengmai 22, Zhengmai 16, Zhonngyu 1211, Luomai 34 and Zhoumai 18). A randomized complete block design was used in all experiments, in which winter wheat was planted in 42 plots (each with a size of 2.6 m × 5 m) with two replicates.

#### Experiment 2

**Fig. 1:**
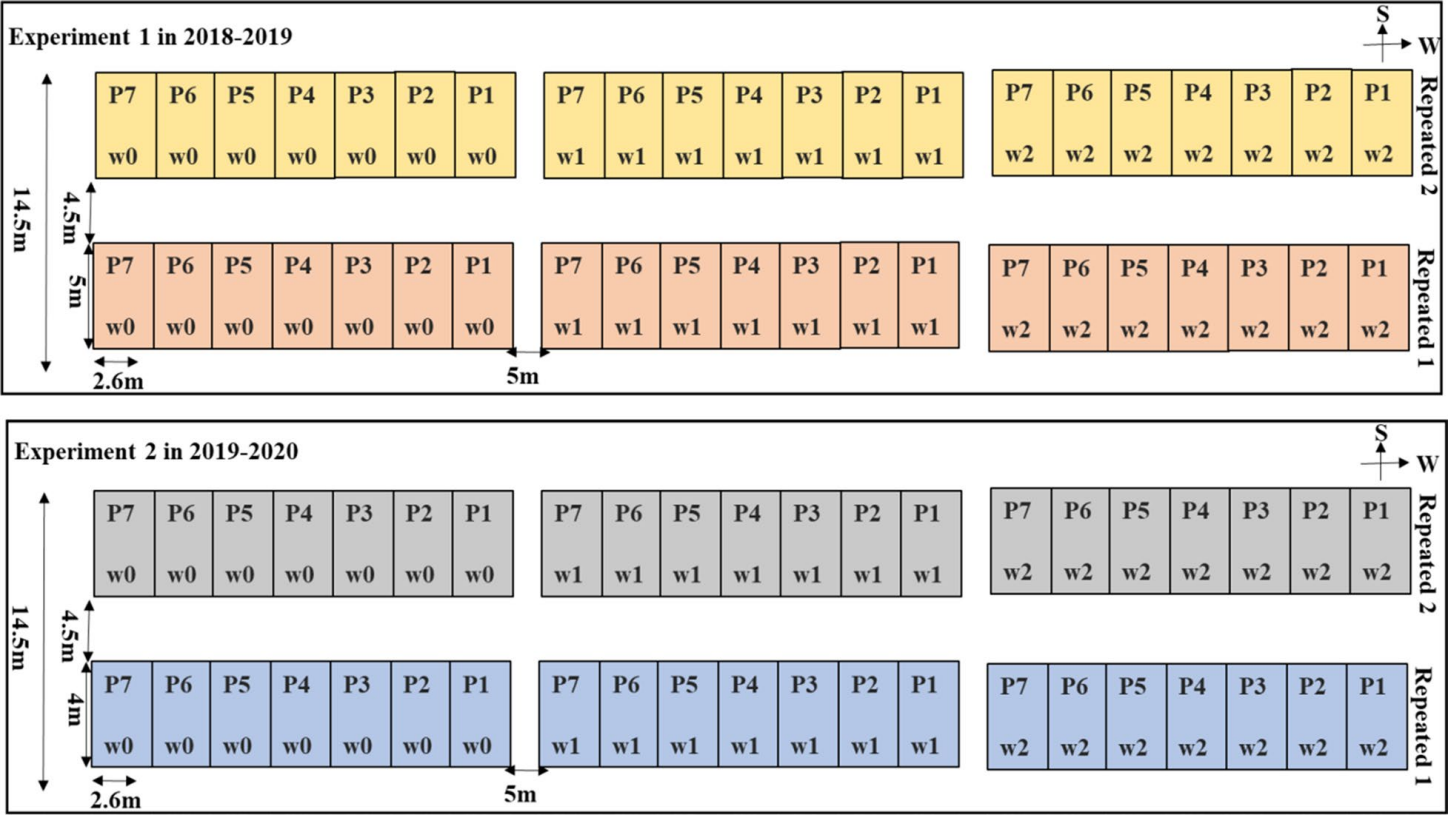
Layout of the experimental design in 2018–2019 and 2019–2020. P1, P2, P3, P4, P5, P6 and P7 represented wheat cultivars, P1: Luomai 27, P2: Zhengmai 136, P3: Zhengmai 22, P4: Zhengmai 16, P5: Zhongyu 1211, P6: Luomai 34, P7: Zhoumai 18. w0, w1, and w2 represent different irrigation treatments: w0 is the irrigation during the bottom stage, w1 is the irrigation during the bottom and jointing stages, and w2 is the irrigation during the bottom, jointing, and grain-filling stages

The experimental site was the same as that of experiment 1 (112° 49′ 18″ E, 36° 64′ 14″ N from 2019 to 2020). The soil type in this area is loamy cinnamon soil (Organic Matter: 13.2 g kg^−1^, Total N:1.05 g kg^−1^, P_2_O_5_:18.6 mg kg^−1^, K:116.3 mg kg^−1^). The area of the sub-region was 10.4 m^2^ (2.6 m × 4 m). The cultivation management measures and the sampling period were the same as those in experiment 1. Additional details regarding the experimental design and the sample collection time are presented in Table 1 and Fig. 1.

**Table 1:** Experimental treatments and sampling periods

Exp.no.	Year	Cultivars	Irrigation treatments	Irrigation date	Sampling time and date
Exp. 1	2018–2019	Luomai 27 Luomai 34 Zhengmai 16 Zhongyu 1211 Zhengmai 136 Zhoumai 18 Zhengmai 22	w0	Irrigation at the bottom stage/October 18, 2018	Mar 15
			w1	Irrigation at the bottom stage/October 18, 2018	Mar 30
				Irrigation at the jointing stage/March 20, 2019	Apr 18
			w2	Irrigation at the bottom stage/October 18, 2018	Apr 25
				Irrigation at the jointing stage/March 20, 2019	May 10
				Irrigation at grain-filling stage/May 1, 2019	May 20
Exp. 2	2019–2020	Same as above	W0	Irrigation at the bottom stage/October 22, 2019	Mar 13
			W1	Irrigation at the bottom stage/October 22, 2019	Mar 28
				Irrigation at the jointing stage/March 18, 2020	Apr 15
			W2	Irrigation at the bottom stage/October 22, 2019	Apr 23
				Irrigation at the jointing stage/March 18, 2020	May 8
				Irrigation at grain-filling stage/April 25, 2020	May 18

### Determination method and index

#### Measuring plant hyperspectral data

All canopy spectral reflectance measurements were obtained from a height of 1.0 m above the vertical canopy under clear sky conditions between 10:00 and 14:00 (Beijing local time), using ASD Field Spec Pro spectrometer (Analytical Spectral Devices, Boulder, CO, USA) with a 25° field of view fiber optics. Reflectance values in the 350–2500 nm range were collected with a sampling interval of 1.4 nm and spectral resolution of 3 nm between 350 and 1050 nm, and 2 and 10 nm, respectively, between 1050 and 2500 nm. Moreover, the black and baseline reflectance was calculated using a 0.40 × 0.40 m^2^ white calibration panel made of BaSO4.To minimize the effects caused by sky and field conditions, spectral measurements were obtained from 10 sites in each plot and averaged into a single spectral sample. For each experiment, measurements were obtained on several dates that reflected the major growth stages of wheat. Using ViewSpec Pro version 6.0 software to assemble and interpret date.

#### Determination of the leaf water content

After the canopy spectra was measured, wheat plants were collected at corresponding points, and all leaves were extracted. The water content of the wheat leaves was determined by the drying method. The fresh weight of the leaves was weighed with an analytical balance (accuracy of 0.01 g). The leaves were then placed into an oven at 105 °C for 30 min. Finally, the samples were overdried at 80 °C until a constant weight was reached, which took approximately 3–5 days, and the dry weight was recorded. The LWC was calculated using the following formula [16]:

1 $$\text{LWC}=\frac{({\text{m}}_{\text{f}}-{\text{m}}_{\text{d}})}{{\text{m}}_{\text{f}}}\times 100$$

where LWC is the water content of leaf, g.g^−1^; ${\text{m}}_{\text{f}}$ is the fresh weight of leaves, g; ${\text{m}}_{\text{d}}$ is the dry weight of the leaves, g.

### Data analysis and utilization

**Fig. 2:**
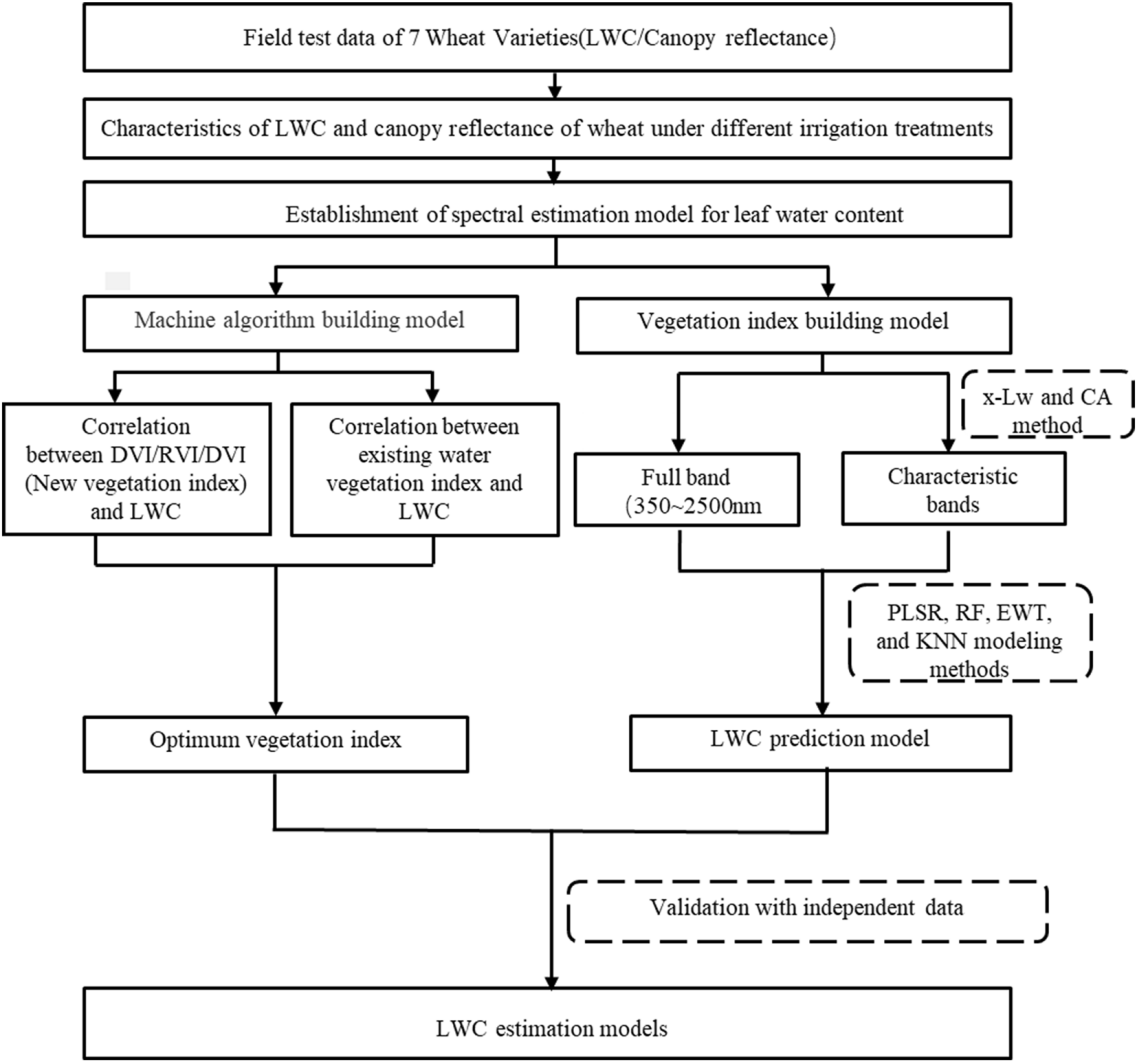
Flow chart of the overall research work performed

During the 2 years of the experiment, a total number of 252 wheat samples were collected. The data from 2018–2019 were used for model calibration (n = 126), whereas the test data from 2019–2020 were utilized as validation samples (n = 126). The statistical parameters of the leaf water content determined in each sample set are shown in Table 2. A flow chart of the development and application of the winter wheat LWC estimation modeling method can be seen in Fig. 2.

**Table 2:** Statistical parameters of the leaf samples used for determination of wheat leaf water content (LWC %)

Sample sets	Experimental year	Sample size	Maximum		Minimum	Mean	Standard deviation	Coefficient of variation
Total	2018–2020	252	88.61		55.87	78.82	4.76	6.02
Modeling set	2018–2019	126	88.61		55.87	78.80	5.71	7.25
Validation set	2019–2020	126	83.75		67.54	78.84	3.92	4.98

#### Characteristic band screening

Due to the effect of the atmosphere on reflectance measurements, the ranges 1350–1400, 1800–1950, and 2450–2500 nm were excluded in this study. The assessment of the input of all spectral bands in the model would cause "dimension disaster". Therefore, the correlation coefficient and the load factor methods are used to screen the characteristic bands.

1. Correlation coefficient (CA)

This method determines the characteristic bands based on a coefficient of the correlation between the spectral band and the parameters. The correlation between the leaf water content and the canopy reflectance under different irrigation treatments was analyzed in this investigation. The characteristic wavelength was determined by selecting the maximum absolute value of the correlation coefficient and the position of the wave crest and trough.

(2) x-Loading weight (x-Lw)

The loading weight based on the PLSR model can establish the proportion of the influence of different dependent variables of that of the total independent variables, which is of great significance for the rapid screening of characteristic bands [17]. The peak and valley values are extremum and can reflect the influence of a specific waveband on water content. In this study, the peak and trough were selected as the characteristic bands.

Based on the feature bands selected by the aforementioned two methods as independent variables, the inversion models for leaf water content estimation of winter wheat were constructed by PLSR, RFR, ERT, and KNN.

#### Modeling method

1. Spectral index

In line with the existing research, the spectral index related to crop water status has been selected as shown in Table 3, and the relationship with leaf water content has also been analyzed. To obtain better spectral parameters, the normalized vegetation index (NDVI), the ratio vegetation index (RVI) and the difference vegetation index (DVI) were calculated in the range of 350–2500 nm, which were shown in the following formula, and the relationship between them and leaf water content was analyzed, so as to determine the optimal spectral estimation of leaf water content parameters were measured.

2 $${\text{NDVI }}\left( {\lambda {1}, \, \lambda {2}} \right) = \left( {{\text{R}}\lambda {1} - {\text{R}}\lambda {2}} \right)/ \, \left( {{\text{R}}\lambda {1} + {\text{ R}}\lambda {2}} \right)$$

3 $${\text{RVI }}\left( {\lambda {1}, \, \lambda {2}} \right) = {\text{ R}}\lambda {1}/{\text{R}}\lambda {2}$$

4 $${\text{DVI }}\left( {\lambda {1}, \, \lambda {2}} \right) = {\text{ R}}\lambda {1} - {\text{R}}\lambda {2}$$

**Table 3:** The calculation method and reference of spectral index related to crop moisture status

Spectral index	Definition or equation	References
Ratio Index	R1650/R2220	Elvidge et al. [18]
Normalized differential water index, NDWI	(R860−1240)/(R820 + 1240)	Gao et al. [19]
Moisture stress index, MSI	R1600/R820	Hunt et al. [20]
Maximum water difference index, MDWI	(Rmax1500−1750)-(Rmin1500−1750)/(Rmax1500−1750) + (Rmin1500−1750)	Eitel et al. [21]
Hyperspectral normalized difference vegetation index, hNDVI	(R900−R680)/(R900−R680)	Rouse et al. [22]
Water index, WI	R900/R970	Penuelas et al. [23]
Simple ratio water index, SRWI	R820/R1200	Zarco-Tejada et al. [24]
Normalized Difference Infrared Index, NDII	(R820−R1649)/(R820 + R1679)	Hardisky et al. [25]
WI/hNDVI	(R900/R970)/[(R900−R680)/(R900−R680)]	Zhang et al. [8]
FD730-955	FD730/FD955	Liang et al. [26]

R λ 1 and R λ 2 represent the reflectivity of any two bands in the range of 350–2500 nm, respectively, and were selected by using self-developed code in MATLAB software.

(2) Partial Least-Squares Regression (PLSR)

A model can be effectively established by PLSR in cases of significant multiple correlations of independent variables [27]. In the process of modeling, the large number of measured collinear spectral variables was reduced to a few non-correlated latent variables [14]. Therefore, the cross-correlation among multiple hyperspectral features can be explained by this method [28]. Here, we calculated and used the number of latent vectors yielding the smallest root mean squared error. We determined the number of latent vectors by the "Loo" cross-validation method using the Python 2.7 package “sklearn.cross decomposition”.

(3) Random Forest Regression (RFR)

RFR can establish the relationship between multiple independent variables and a dependent variable. Importantly, this method improves the prediction accuracy of the individual model through fitting many regression trees [29]. To apply this technique, we used “sklearn. tree” in Python 2.7, with “ n_estimators = 500, max_features = ‘sqrt’”.

(4) Extreme Random Trees (ERT)

ERT is a top-down method that is very similar to the random forest approach, but is different from the latter in two points: first, it does not adopt bootstrap sampling with a replacement strategy but directly uses the original training samples to reduce the deviation; second, the bifurcation value is completely random, which can achieve the bifurcation of a decision tree. The result is smaller and more stable than that of the random forest [30]. To apply this technique, we used library – “sklearn. ensemble” in Python 2.7, with the parameters of “n_estimators = 500, max_features = ‘sqrt’”.

(5) K-Nearest Neighbor (KNN).

KNN was proposed by Cover and Hart, which is a classification algorithm based on the proximity of similar samples in the pattern space [31]. Euclidean distance is used to measure the similarity between samples. A larger distance lowers the similarity. In this study, the k-nearest neighbor algorithm was employed in “sklearn. neighbors” of Python 2.7. A cross-validation method was used to determine K-values, K = 3.

### Model validation

The coefficient of determination (R^2^), root mean square error (RMSE) and the ratio of performance to deviation (RPD) were used to evaluate the accuracy of the model. The calculation formula is as follows:

5 $${R}^{2}=1-\sum_{i=1}^{n}{\left({y}_{i}-{\widehat{y}}_{i}\right)}^{2}/\sum_{i=1}^{n}{\left({y}_{i}-{\stackrel{-}{y}}\right)}^{2}$$

6 $$\text{RMSE}=\sqrt{\frac{1}{n}\times \sum_{i=1}^{n}{({y}_{i}-{\widehat{y}}_{i})}^{2}}$$

7 $$\text{RPD}=SD/RMSE$$

where y and ${\widehat{y}}$ represent the measured and predicted values, respectively. $\overline{y}$ represents the average of measured values. n is the number of samples. SD is the standard deviation of the measured data. The larger R^2^ with smaller RMSE values indicate good model prediction accuracy. The RPD values > 1.4 indicate that the prediction ability of the model is acceptable, and the model can be applied.

## Results

### Effects of the irrigation times on the leaf water content (LWC) and canopy spectral reflectance of wheat

**Fig. 3:**
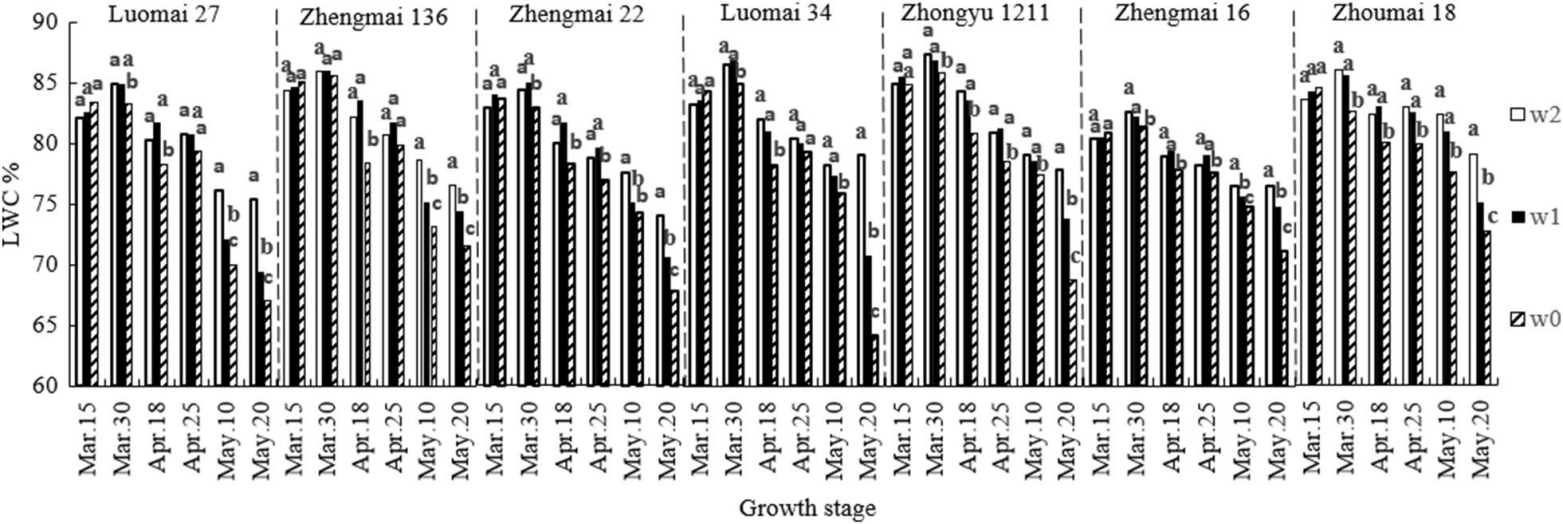
Effects of different irrigation times on water content of wheat leaves. The letters on the column indicate the significant difference among different treatments in the same period (*P* < 0.05)

To explain the effects of different irrigation times on LWC and canopy spectra, the data of experiment 1 were taken as an example. As can be seen from Fig. 3, plant growth of all varieties was accompanied with an initial increase in LWC, followed by a decrease. The highest LWC value was reached 10 days after the irrigation at the jointing stage (Mar 30). At the early wheat growth stage, an insignificant difference was observed in LWC among the different irrigation time treatments. However, it decreased rapidly in the late growth period, which gradually increased the difference. The LWC in w0 was the lowest, which was significantly lower than those in w2 and w1. Our results showed that the irrigation at the grain-filling stage delayed leaf senescence and prolonged the green color-retaining period of leaves.

**Fig. 4:**
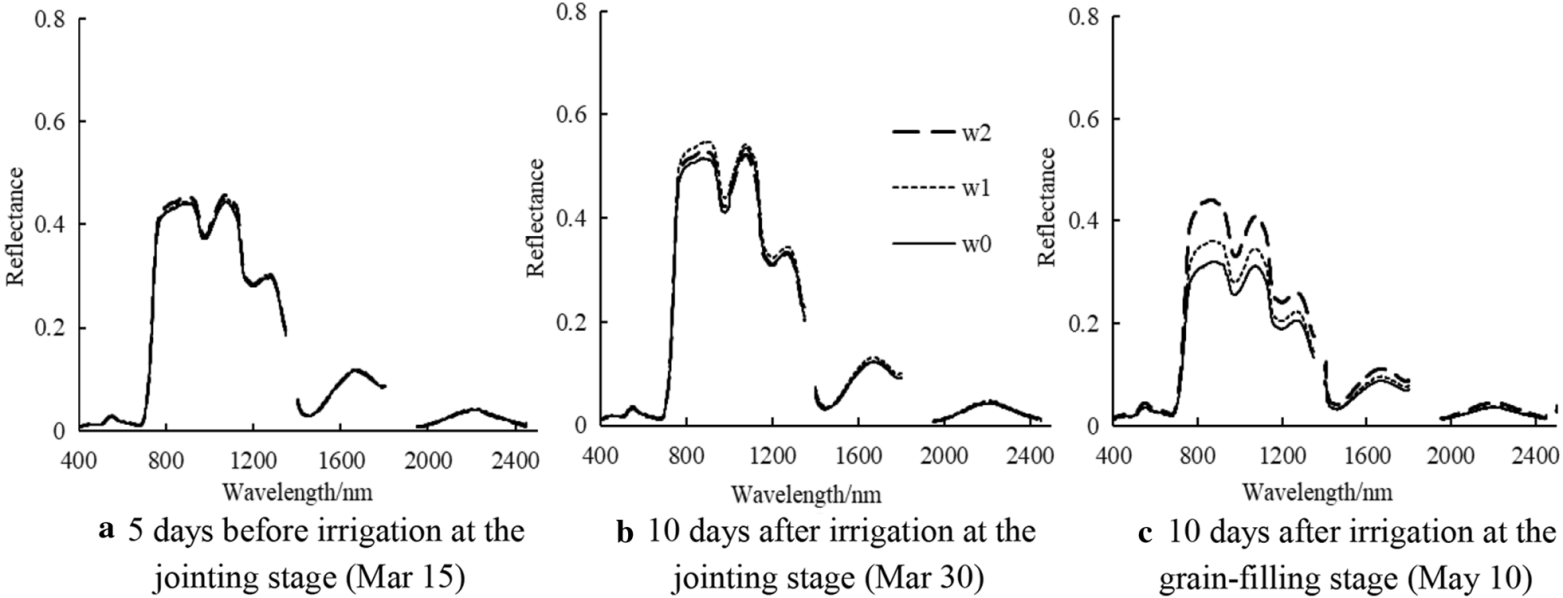
Spectral characteristics of LWC under different irrigation times

We analyzed the effect of irrigation times on wheat spectral reflectance in Luomai 27, considering it a representative example (Fig. 4). Five days before the irrigation at the jointing stage (Mar 15), the canopy reflectance was slightly lower than that detected 10 days after the irrigation at the jointing stage (Mar 30) at the same irrigation treatment times. The reflectance decreased again after the point of 10 days after the irrigation at the grain-filling stage (May 10). Therefore, the canopy reflectance was initially augmented but then decreased with the advancement in growth. The canopy reflectance in the visible region (350–750 nm) did not change significantly with the prolongation in the irrigation times, whereas the canopy reflectance in the near-infrared region (750–1350 nm) gradually rose. The main reason for that tendency is that the sufficient water supply received by plants through the irrigation accelerated the growth of leaf area index and biomass. In return, the canopy reflectance increased. In particular, there was significant difference in canopy reflectance among different irrigation treatments after filling water, the reflectance of w2, w1, and w0 reached 43.45%, 35.28%, and 31.01% at 820 nm, respectively. The values of the canopy reflectance of w2, w1, and w0 were 32.27%, 28.08%, and 25.58% at 970 nm, respectively. The canopy reflectance of w2, w1, and w0 were 24.00%, 20.47%, and 18.85% at 1266 nm, respectively. It is more evident after filling water, that was w2 > w1 > w0. The different irrigation times did not influence the variations in the canopy spectral reflectance, which were basically the same in all wheat varieties.

### Correlation between LWC and spectral index of wheat leaves

**Fig. 5:**
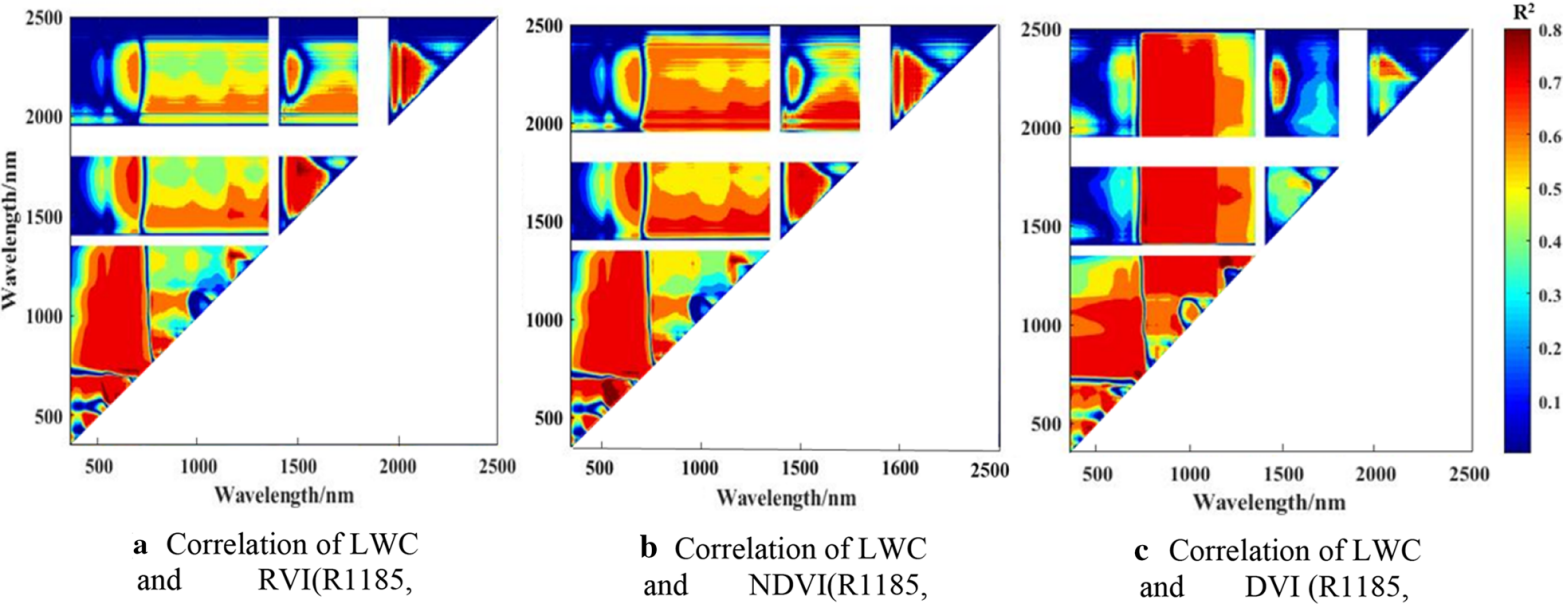
Contour maps of the coefficients of determination (R^2^) between LWC and RVI, NDVI and DVI values based on canopy spectra

To establish a new spectral vegetation index, the correlations between LWC and all possible two-band combinations of NDVI (normalized spectral index), RVI (ratio spectral index, and DVI (difference spectral index) were analyzed. Then, the contour maps of coefficient of determination between LWC and new spectral vegetation index were plotted (Fig. 5). According to Fig. 5, the highest R^2^ band combination was extracted from the hot spot area as the best spectral index of leaf water content. The results showed that the sensitive regions of the three spectral index combinations were consistent, and the combination of 800–1300, 1600–1900, and 1950–2200 nm was better. The best result was obtained that NDVI, RVI, and DVI consisting of 1185 nm and 1307 nm performed the best for LWC estimation.

**Fig.6:**
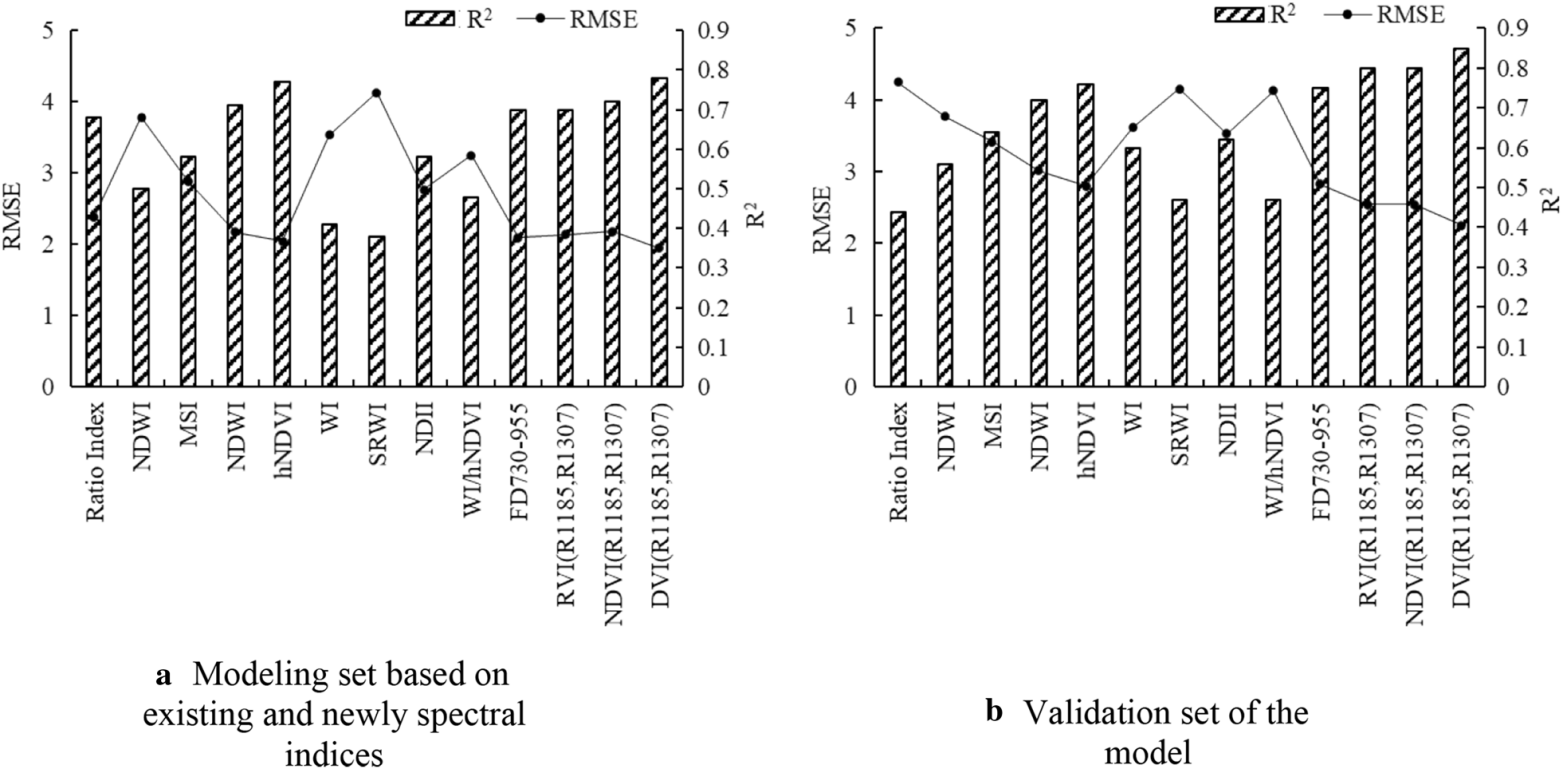
Coefficient of determination between 13 spectral indices and LWC

**Fig.7:**
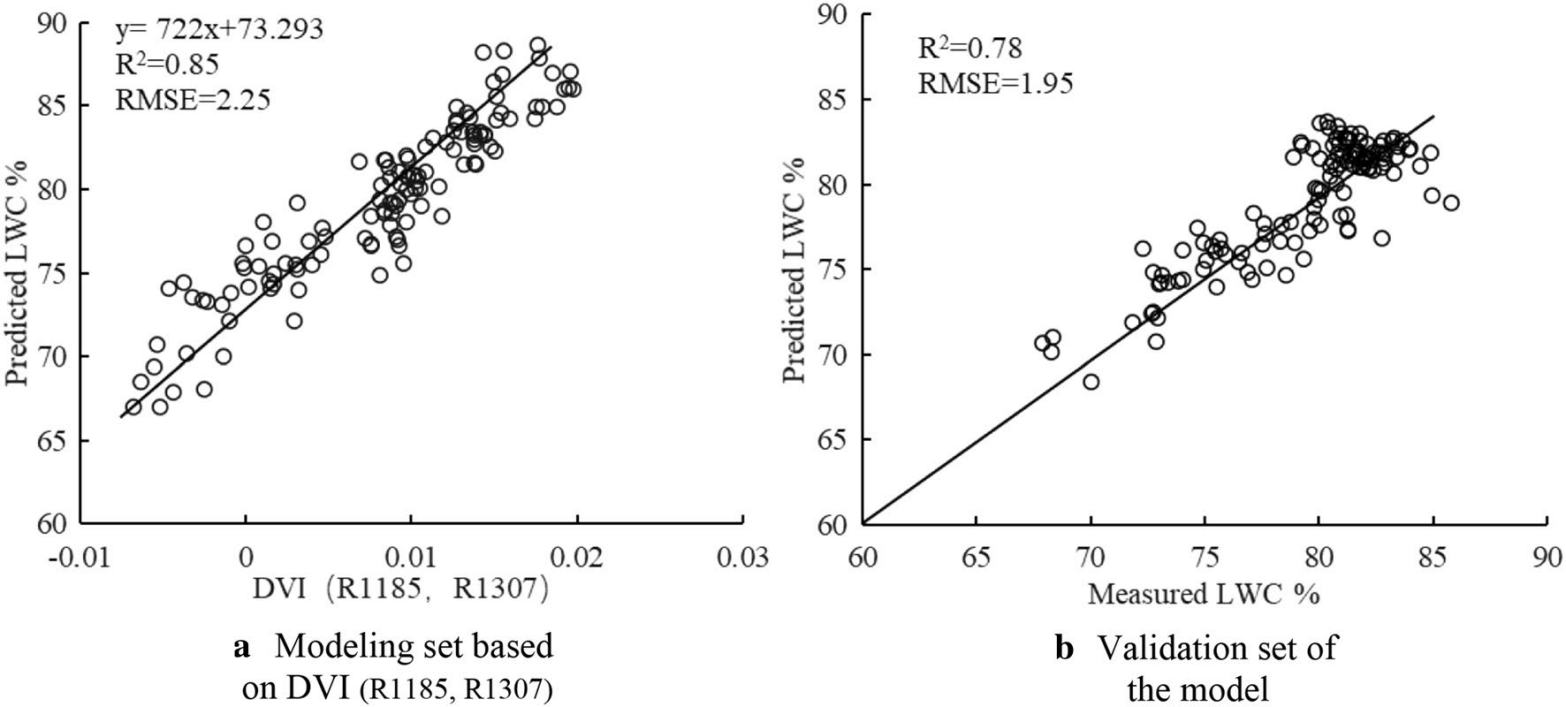
Relationships between leaf water content (LWC) and spectral index DVI (R1185, R1307) and model verification

Analyses of the correlations between wheat leaf water content under different irrigation treatments and the existing water-related vegetation indexes (Ratio index, NDWI, MSI, MDWI, hNDVI, NDII, WI, SRWI, WI/hNDVI, and FD_730-955_), as well as of the newly developed three vegetation indexes were conducted, and the prediction performance of the models constructed by the 13 spectral indices were compared and analyzed (Fig. 6). In the modeling set, MSI, NDWI, hNDVI, WI, NDII, and FD_730–955_ were the spectral indices with R^2^ higher than 0.6. The models were validated by the data obtained in Experiment 2, where R^2^ ranged from 0.38 to 0.78. Our results revealed that the proposed DVI (R1185, R1307) had the best performance with high R^2^ and low RMSE values. The best linear equation of LWC predicted by DVI value was illustrated in Fig. 7, with calibration R^2^ of 0.85 and RMSE of 2.25, validation R^2^ of 0.78, and RMSE of 1.95. Therefore, the newly developed indices can be used for accurate estimation of the changes in RWC caused by irrigation times in wheat.

### Extraction of the characteristic bands of LWC

**Fig.8:**
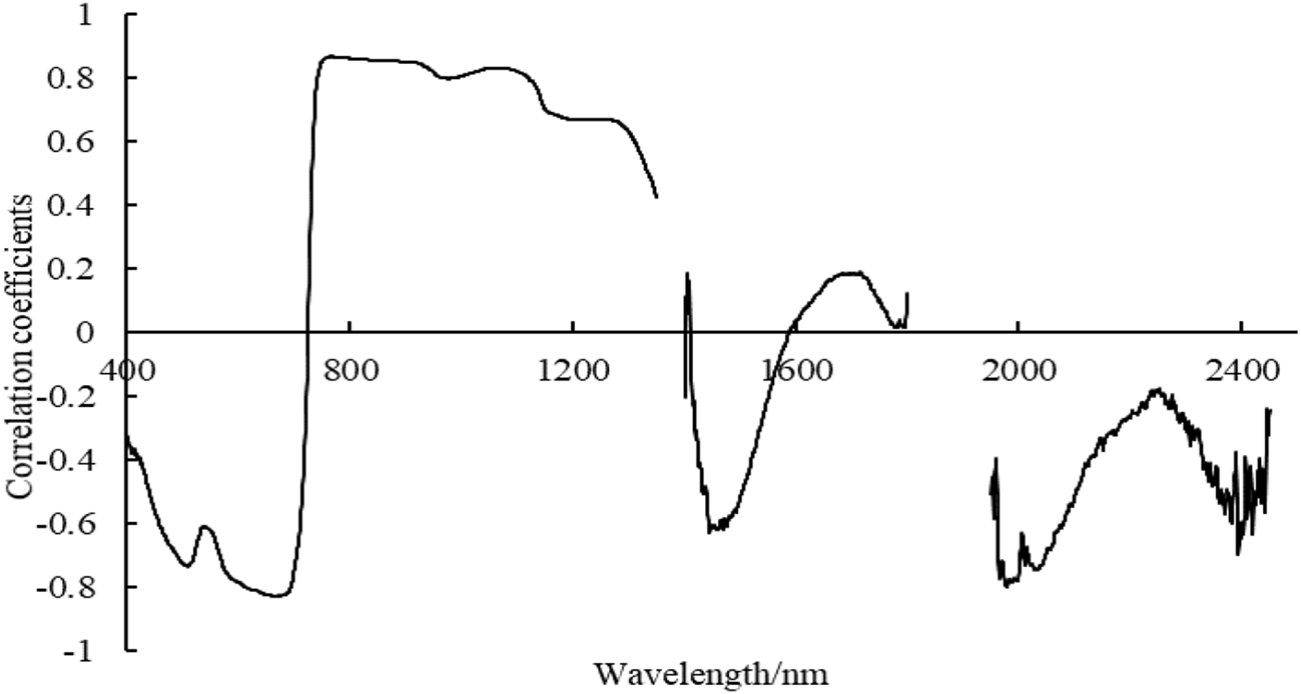
Correlation between canopy reflectance and leaf water content (LWC)

The correlation between LWC and the original spectral reflectance (350–2500 nm) under different irrigation times was depicted in Fig. 8. We found that the correlation coefficient ranged from −0.83 to 0.87, with maximum positive and negative correlation coefficient values of 0.86 (618 nm) and −0.83 (769 nm), respectively. By selecting the maximum absolute value of the correlation coefficient at the local peaks and troughs, we established the optimal wavelengths, which were 505, 551, 681, 747–831, 989, 1101, 1158, 1445, 1716, 1782, 1978, 2000, 2007, 2038, 2242, and 2394 nm.

**Fig. 9:**
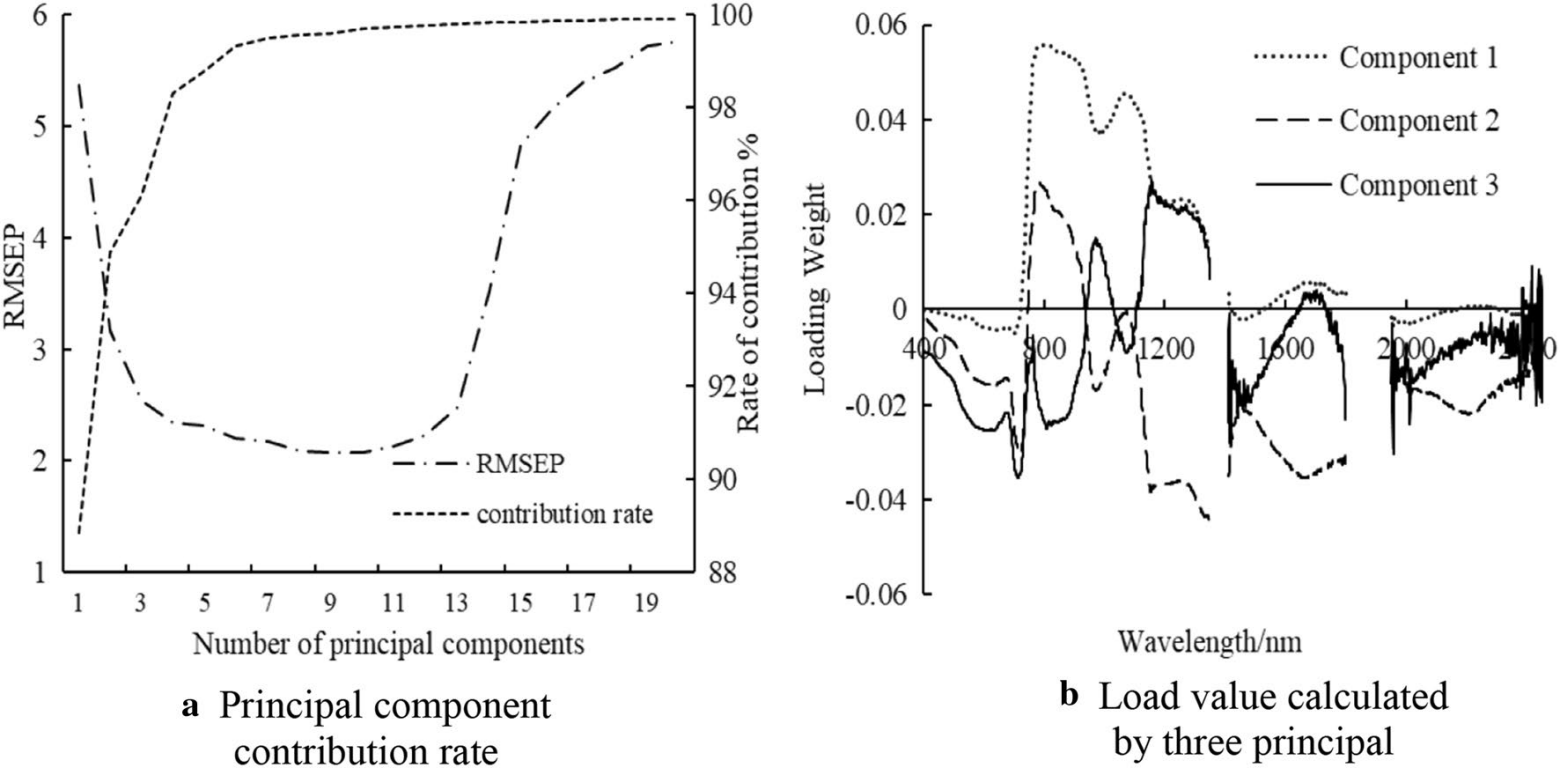
Principal component contribution rate and load value calculated by PLSR regression

Based on the contribution rate of the PLSR model and RMSEP, the number of principal components was determined. When the principal component was 3, RMSEP was 2.34% and explained 96.12% of the variance (88.86%, 6.03%, and 1.23% for PC1, PC2, and PC3, correspondingly), which was presented in Fig. 9a. Therefore, the best characteristic band was determined by the peaks and troughs of the loading weight values of the three principal components (Fig. 9b). According to the data if the above analysis, the optimal bands determined were 588, 663, 674, 680, 700, 763, 777, 783, 808, 816, 970, 977, 984, 1070, 1072, 1156, 1205, 1246, 1264, 1402, 1445, 1456, 1660, 1678, 1957, 1702, 2221, and 2252 nm.

### Comparison of LWC inversion models constructed by different methods

The full band and the characteristic band selected by correlation coefficient (CA) and x-Loading weight (x-Lw) were used as independent variables respectively, and LWC was taken as the dependent variable. After the PLSR, KNN, RF and ERT models were established.

**Fig.10:**
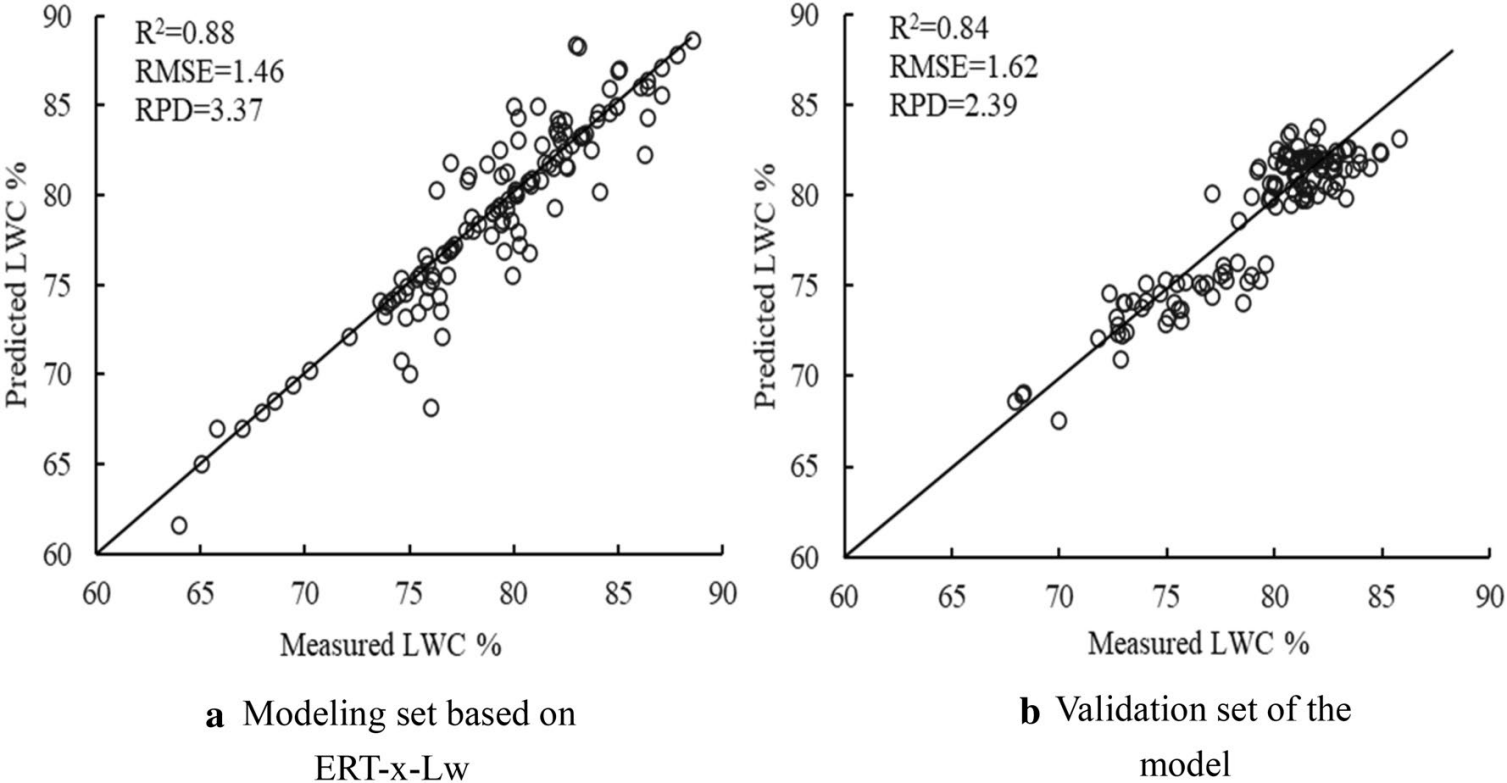
Modeling and validation results of leaf water content (LWC) and characteristic bands based on ERT-x-Lw

The full and characteristic bands that were selected by the two methods as input and the models constructed by different modeling methods are presented in Table 4. The accuracy of the model was not significantly lower than that of the full-band model, with feature bands extracted from CA and x-Lw as independent variables, but the input variables were reduced, which improved the efficiency of the model. For 100 characteristic bands selected by CA method was used as the input of the model, and the performance of the model was as follows: PLSR-CA > ERF-CA > RF-CA > KNN-CA. For the 28 characteristic bands selected by x-Lw method, the performance of the model was as follows: ERF-x-Lw > PLSR-x-Lw > RF-x-Lw > KNN-x-Lw. Compared with the CA method, the number of the independent variable was lower by 98.63% in the x-Lw method, which significantly improved the modeling efficiency. Among the 12 models constructed, the ERT-x-Lw model had the higher R^2^ value and lowest RMSE. In the modeling set, R2, RMSE, and RPD were 0.88, 1.46, and 3.37, respectively, whereas in the validation set, R2, RMSE, and RPD were 0.84, 1.62, and 2.39, correspondingly (Fig. 10).

**Table 4:** Regression analysis of the characteristic bands and LWC by different modeling methods

Modeling method	Feature band screening method	Number of modeled bands	Modeling			Validation		
			R^2^	RMSE	RPD	R^2^	RMSE	RPD
PLSR	Full band	1901	0.87	2.11	2.73	0.82	1.78	2.09
	CA	100	0.86	2.10	2.52	0.84	1.61	2.43
	x-LW	28	0.86	2.61	1.96	0.84	1.73	2.04
RF	Full band	1901	0.88	2.80	1.26	0.83	1.69	2.31
	CA	100	0.90	2.49	1.63	0.81	1.75	2.15
	x-LW	28	0.88	1.57	3.10	0.80	1.86	1.92
ERF	Full band	1901	0.87	1.36	2.18	0.85	1.52	2.30
	CA	100	0.86	1.95	2.48	0.82	1.76	2.34
	x-LW	28	0.88	1.46	3.37	0.84	1.62	2.39
KNN	Full band	1901	0.82	2.10	1.61	0.83	1.61	2.20
	CA	100	0.85	2.00	2.25	0.80	1.79	2.16
	x-LW	28	0.84	2.04	1.80	0.80	1.74	1.83

## Discussion

The growth and development of wheat can be directly affected by water deficit. Leaf water content is an important indicator of wheat growth that can be monitored by hyperspectral technology. The hyperspectral reflectance technique, which detects the spectral reflectance of the canopy from visible- (VIS) to shortwave-infrared (SWIR), has been shown to have a great potential to detect even the slight variations and modifications in biophysical and biochemical characteristics of crop canopy [32, 33]. Furthermore, canopy reflectance in the near-infrared (NIR,700–1300 nm) and the shortwave-infrared (SWIR, 1300–2500 nm) regions are strongly influenced by several internal leaf structural and water content of the canopy. Therefore, it is successfully used to track the changes in plant parameters related to plant water status and monitor the changes of biomass and leaf area indirectly caused by wheat moisture [34]. In this study, the spectral reflectance of wheat canopy initially increased but then decreased with the advance of plant growth. At the jointing stage, the canopy reflectance of wheat increased after the irrigation was performed, compared with that without irrigation. Further, the reflectance decreased significantly in the treatment of 10 days after the irrigation at the grain-filling stage. The reason for these results may be that a growth of wheat biomass and leaf area at the jointing stage, especially the timely irrigation at that stage, was able to increase the water absorption rate of plants, elevating the leaf water content and thus further accelerating the growth of wheat plants, which ultimately contributed to improving the canopy reflectance. However, at the later stage of grain-filling, the leaf area and leaf water content gradually decreased with plant senescence, which led to a decline in canopy reflectance [35, 36].

In addition, the different irrigation times exerted a significant effect on canopy spectral reflectance. No significant difference was observed among three treatments before and after the jointing stage. In the near-infrared region (750–1350 nm), the canopy reflectance increased significantly with the increase of the irrigation times after the jointing stage, which was due to the rise in the wheat plant height, chlorophyll content, and net photosynthetic rate. It is noteworthy that the canopy reflectance of w2 was significantly higher than those of w1 and w0. This result indicates that the leaf senescence and photosynthesis time after flowering can be delayed by irrigation at the grain-filling stage. However, without irrigation, wheat plants grow shorter; the leaves turn yellow and wither ahead of time; the lower leaf water content causes cell structure changes, which eventually leads to wheat yield reduction [36].

The change of canopy reflectance is caused by the change of LWC. In this study, LWC showed no significant differences among the treatments on Mar 15 (Fig. 3) and canopy reflectance also had no changes (Fig. 4a). At 10 days after the irrigation at the jointing stage, the LWC of the non-irrigated treatment (w0) was significantly lower than those of the other treatments (Fig. 3), and the canopy reflectance was the lowest in the near-infrared band (Fig. 4b). After the irrigation at the grain-filling stage, LWC was marked by significant differences among the treatments (w2 > w1 > w0; Fig. 3), and canopy reflectance also changed (w2 > w1 > w0; Fig. 4c). A previous study established water-sensitive bands at 820, 970, 1200, and 1450 nm [37]. In this study, we found that canopy reflectance in these bands was different among the treatments, and decreased with the decline of LWC. Therefore, our model was constructed by the characteristic response band of leaf water content, which can be used to diagnose and retrieve leaf water content. Many studies have developed spectral indices for estimating of the leaf water index. However, due to the diversity in the experimental conditions in different studies, various bands have been selected in the spectral index. For example, Liang et al. [26] used the first derivative ratio index at 730 and 955 nm to predict wheat leaf water content, achieving a value of the modeling prediction coefficient R^2^ of 0.74. In another investigation, Jiang et al. [38] selected the bands at 1300 and 1200 nm for the development of a ratio spectral index for the prediction of the water content in wheat leaves, with R^2^ of 0.63. On the other hand. The bands at 1391 and 1830 nm were used by other researchers to predict the wheat leaf water status [9]. DVI (R1185, R1307) developed in this study showed high accuracy; moreover, it is similar to wavebands selected by Jiang et al. [38] and Wu et al. [39]. Furthermore, the two bands selected in this study were in the water-sensitive near-infrared region [40]. Our model yielded a modeling R^2^ value of 0.85, and a prediction R^2^ value of 0.78 and was thus superior to the water spectral indices developed in previous studies.

To improve the modeling accuracy, machine learning and other methods have also been applied to model and analyze the water content of wheat. Several researchers in the past [41], based on the grey correlation analysis method, selected a spectral index with a high correlation to be used for leaf water content analysis. These spectral indices were used as independent variables in PLSR and Back Propagation (BP) neural network models to predict wheat leaf water content, with R^2^ values of 0.72 and 0.80, respectively [41, 42]. In the present study, the correlation coefficient (CA) and x-loading weight (x-Lw) methods were employed to select the characteristic bands. Compared with the CA method, the x-Lw method reduces the number of bands by 72%, which may be due to the concentration of sensitive bands extracted by the CA method and the smaller adjacent interval [43]. Twenty-eight characteristic bands related to leaf water content were selected by x-Lw. Among them, the wavelengths of 663, 674, 680, and 700 nm are located in the "red edge" region, which can indirectly diagnose the water status of wheat due to the high reflection of the crop leaf structure [44]. On the other hand, 1156, 1205, 1246, and 1264 nm are related to the leaf and canopy cell structure [12]. Additionally, 1402, 1445, 1456, and 1957 nm are associated with the water absorption band [45]. This is basically consistent with the water-related bands selected in a previous experiment [12]. Among the four modeling methods, the one using x-Lw-ERT provided the best prediction for leaf water content retrieval. The values of the coefficient of determination (R^2^) during the calibration and validation were 0.88 and 0.84, correspondingly, and RMSE were 1.46 and 1.62, respectively which were higher than those of the PLSR, RF, and KNN models. These outcomes were probably due to that the better generalization ability and more stable performance of ERT [46]. However, KNN breaks the continuous characteristics of the band because it learns and predicts according to the distance features between different samples [47]. Our findings suggest that the extreme random tree (ERT) may be a reliable modeling method to improve the modeling accuracy of machine learning and other methods used to model and analyze the moisture content of wheat.

## Conclusion

We studied the effect of different irrigation times on the wheat canopy reflectance spectrum based on a field experiment with different irrigation treatments in two consecutive years. Five different models were compared, the results of which showed that the irrigation at the jointing + grain-filling stage increased the leaf water content, leaf area, and biomass; moreover, plant senescence was delayed and canopy reflectance elevated. The new model constructed by DVI (R1185, R1307) can be used to estimate the LWC of winter wheat. The accuracy of the extreme random tree model based on the x-loading weight method had the best performance among the other compared models. Therefore, both models can be used to estimate the water content of wheat leaves effectively.

## Data Availability

The processed data required to reproduce these findings cannot be shared at this time as the data also forms part of an ongoing study.
